# Analysis focusing on plasma von Willebrand factor in pachychoroid neovasculopathy and age-related macular degeneration

**DOI:** 10.1038/s41598-021-99557-6

**Published:** 2021-10-07

**Authors:** Hiromasa Hirai, Mariko Yamashita, Masanori Matsumoto, Masaki Hayakawa, Kazuya Sakai, Tetsuo Ueda, Nahoko Ogata

**Affiliations:** 1 Department of Ophthalmology, Nara Medical University, 840 Shijo-Cho, Kashihara, Japan; 2 Department of Ophthalmology, Nara City Hospital, 1-50-1 Higashikidera-cho, Nara, Japan; 3 Department of Blood Transfusion Medicine, Nara Medical University, 840 Shijo-Cho, Kashihara, Japan

**Keywords:** Diseases, Pathogenesis

## Abstract

Pachychoroid neovasculopathy (PNV) is a new concept of macular disorder. Some cases diagnosed as age-related macular degeneration (AMD) have been re-diagnosed as PNV. However, the biological features of PNV are still uncertain. The purpose of this study was to compare PNV and AMD by analyses focusing on von Willebrand factor (VWF) and complement factor H (*CFH*). Ninety-seven patients who were previously diagnosed with treatment naïve AMD were enrolled in this study. They were re-classified as either PNV or AMD based on the clinical criteria and 33 patients were classified as PNV and 64 patients as AMD. We examined the clinical data, analyzed VWF multimer and two genetic polymorphisms (I62V and Y402H) in the *CFH*. PNV group was significantly younger than AMD group (*P* = 0.001). In both I62V and Y402H, there were no significant differences between PNV and AMD while the recessive homozygous (AA) was found only in PNV group in I62V*.* The presence of unusually large VWF multimers (UL-VWFMs) and subretinal hemorrhages were significantly higher in PNV than in AMD (*P* = 0.045, *P* = 0.020, respectively). Thus, the residual UL-VWFMs may result in platelet thrombosis and hemorrhages in the choriocapillaris of PNV. In conclusion, our results suggest the biological differences between PNV and AMD.

## Introduction

Age-related macular degeneration (AMD) is a major retinal disease in the elderly^1^. AMD can be categorized by the stage of the disease. In the early stage of AMD, the abnormalities of retinal pigment epithelium (RPE) and drusen (yellowish extracellular material between Bruch's membrane and RPE) are the clinical signs^2^. The late stage of AMD can be classified into neovascular (exudative) AMD and atrophic (dry) AMD^2^. Choroidal neovascularization (CNV) is pathognomonic for neovascular AMD. Typical neovascular AMD is commonly classified according to the location of the CNV, beneath (Type 1) or above the RPE (Type 2). There are also two specific types in neovascular AMD: polypoidal choroidal vasculopathy (PCV) and retinal angiomatous proliferation (RAP)^1^. PCV is characterized by polypoidal lesions in indocyanine green angiography (ICGA) and has recently been considered a subtype of Type 1 AMD^3^. As for atrophic AMD, atrophied RPE and visible choroidal vessels (geographic atrophy) are distinctive.

AMD is a multifactorial disease, and several causes have been suggested such as the sex of the individual, smoking, inflammation, and genetic factors^2,4^. Complement factor H (CFH) is also a well-known risk factor for AMD^5,6^, and it is activated by various pathways to initiate immune responses. This activation may then cause cell damage^7^. CFH also promotes the degradation of the von Willebrand factor (VWF)^8–10^. VWF, a large glycoprotein with a multimeric mass structure, is exclusively produced by vascular endothelial cells as unusually large VWF multimers (UL-VWFMs) and is secreted into the plasma^11,12^. The UL-VWFMs are cleaved by a disintegrin and metalloproteinase with a thrombospondin type 1 motif, member 13 (ADAMTS13). Although VWF plays an important role in pertinent coagulation, the presence of UL-VWFMs in the plasma is a risk factor for arterial thrombosis^13,14^. Some researchers have reported an elevation of the plasma levels of VWF in treatment naïve AMD patients^15,16^, and we also have demonstrated the higher percentages of existence of UL-VWFMs in patients with AMD compared to that in controls^17^.

Pang et al. have proposed a new disease concept called ‘pachychoroid neovasculopathy’ (PNV)^3,18,19^. PNV is a disorder which is characterized by the development of Type1 CNV secondary to central serous chorioretinopathy (CSC) or pachychoroid pigment epitheliopathy (PPE). CSC occurs mostly in middle aged males and is characterized by choroidal thickening and dysfunction of the RPE which results in the presence of subretinal fluid (SRF) and RPE atrophy^19,20^. PPE is characterized by hyperplasia of the RPE and choroidal thickening without SRF, and it was suggested as an incomplete state or a pre-stage of CSC^18^. In PNV, several clinical features have been noted: choroidal thickening (pachychoroid), dilated choroidal vessels (pachyvessels), increased choroidal vascular permeability, and absence of drusen^3,19^. However, the biological features of PNV are still uncertain.

We consider that many cases diagnosed as AMD may in fact be PNV and that comparisons of the biological characteristics of AMD and PNV have not been well done.

Thus, the purpose of this study is to compare the characteristics of AMD to that of PNV. To accomplish this, we re-examined previously diagnosed AMD cases and separated them into PNV and AMD. We then performed VWF-based clinical and hematological analyses and genetic polymorphisms in the *CFH* to investigate the differences between two ocular disorders.

## Results

The baseline characteristics of patients are available in Table 1. The median age was 77.0 years old and seventy were male (72%). There were 51 past or current smokers (53%). The median central choroidal thickness (CCT) was 217 μm and the median choroidal vessel diameter (CVD) was 144 μm. There was a strong positive correlation between CCT and CVD (r = 0.854, *P* < 0.001, Pearson's correlation coefficient). We also measured the blood type of all patients because it has been reported that VWF in individuals with blood type O is more likely to be cleaved by ADAMTS13^21,22^.

**Table 1:** The baseline characteristics of patients (n = 97).

Age, median (IQR)	77.0 (71.0–83.0)
Sex (male), n (%)	70 (72)
BMI, median (IQR), kg/m^2^	22.1 (19.9–23.6)
**Smoking status, n (%)**	
Never	46 (47)
Past or current	51 (53)
Current	27 (28)
Brinkman Index, median (IQR)	150 (0–1000)
**Blood type, n (%)**	
Type O	30 (31)
Type A	34 (35)
Type B	19 (17)
Type AB	14 (13)
CCT, median (IQR), µm	217 (140–291)
CVD, median (IOR), µm	144 (90–186)
IRF, n (%)	16 (16)
SRF, n (%)	61 (63)
PED, n (%)	26 (27)
PCV, n (%)	42 (43)

*IQR* inter-quartile range, Brinkman Index, daily number of cigarettes multiplied by smoking years, *CCT* central choroidal thickness, *CVD* choroidal vessel diameter, *IRF* intra-retinal fluid, *SRF* subretinal fluid, *PED* pigment epithelium detachment, *PCV* polypoidal choroidal vasculopathy.

Of the 97 total patients, 33 patients (34%) were diagnosed with PNV. The comparisons of patients based on PNV and AMD are summarized in Table 2. The patients in PNV group were significantly younger than those in AMD group (*P* = 0.001), and CCT was significantly thicker in PNV group (*P* < 0.001). CVD was also significantly greater in PNV than in AMD (*P* < 0.001). The rate of positive UL-VWFMs was significantly higher in PNV group than that in AMD group (36% vs 17%, *P* = 0.045), and the rate of subretinal hemorrhage was also significantly higher in PNV group than in AMD group (24% vs 6%, *P* = 0.020). The presence of SRF was significantly higher in PNV group than in AMD group (85% vs 52%, *P* = 0.001). There was no difference in the distribution of the blood type between the two groups.

**Table 2:** The comparison of patients; PNV and AMD.

	PNV (n = 33)	AMD (n = 64)	*P* value
Age, median (IQR)	72.0 (65.0–77.0)	78.5 (74.0–84.0)	0.001^†^
Sex (male), n (%)	24 (73)	46 (72)	1^‡^
BMI, median (IQR), kg/m^2^	22.1 (19.2–23.9)	22.1 (20.0–23.1)	0.82^†^
**Smoking status, n (%)**			
Never	15 (45)	31 (48)	–
Past or current	18 (55)	33 (52)	0.83^‡^
Current	8 (24)	19 (30)	0.64^‡^
Brinkman Index, median (IQR)	80 (0–920)	290 (0–1070)	0.71^†^
**Blood type, n (%)**			
Type O	11 (33)	19 (30)	0.82^‡^
Type A	12 (36)	22 (34)	1^‡^
Type B	6 (18)	13 (20)	1^‡^
Type AB	4 (12)	10 (16)	0.77^‡^
CCT, median (IQR), µm	291 (260–349)	165 (120–214)	< 0.001^†^
CVD, median (IOR), µm	202 (186–236)	101 (81–143)	< 0.001^†^
UL-VWFM, n (%)	12 (36)	11 (17)	0.045^‡^
Subretinal hemorrhage, n (%)	8 (24)	4 (6)	0.020^‡^
IRF, n (%)	4 (12)	12 (19)	0.57^‡^
SRF, n (%)	28 (85)	33 (52)	0.001^‡^
PED, n (%)	9 (27)	17 (27)	1^‡^
PCV, n (%)	18 (55)	24 (38)	0.13^‡^

*PNV* pachychoroid neovasculopathy, *AMD* age related macular degeneration, *IQR* inter-quartile range, *CCT* central choroidal thickness, *CVD* choroidal vessel diameter, *IRF* intra-retinal fluid, *SRF* subretinal fluid, *PED* pigment epithelium detachment, *PCV* polypoidal choroidal vasculopathy.

^†^Mann–Whitney U test.

^‡^Fisher’s exact test.

**Figure 1:**
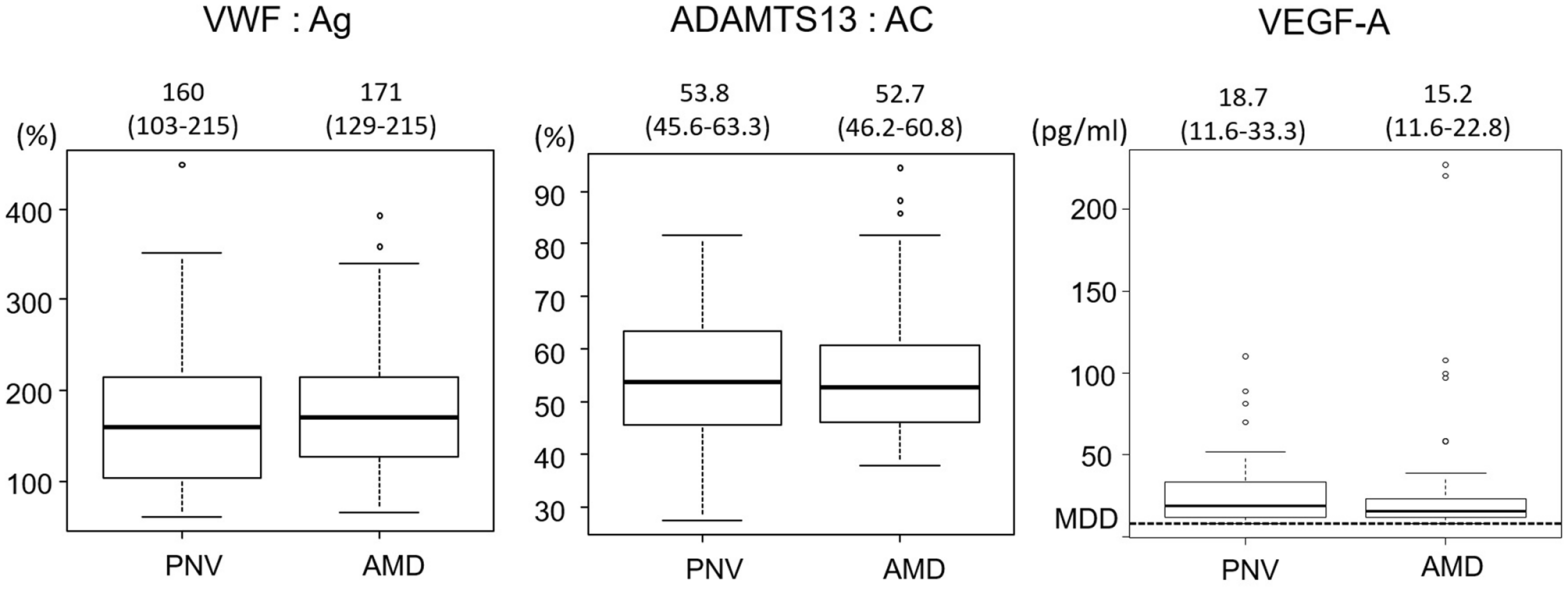
VWF: Ag, ADAMTS13:AC, and VEGF-A in PNV and AMD. Each of the values in the graph is the median with the interquartile range. VWF: Ag, plasma VWF: Antigen. ADAMTS13: AC, plasma ADAMTS13 activity. VEGF-A, vascular endothelial growth factor A. MOD, minimum detectable dose. the MDD of VEGF-A defined by the manufacturer was 9 pg/ml.

The significant differences were not found in the levels of plasma VWF Antigen (VWF: Ag), the activity of ADAMTS13 (ADAMTS13: AC), and the plasma vascular endothelial growth factor A (VEGF-A) between PNV group and AMD group (Fig. 1).

The comparisons of patients based on the presence of UL-VWFMs are summarized in Table 3. The rate of past or current smokers was significantly higher in UL-VWFM positive group than that in UL-VWFM negative group (*P* = 0.032). The frequency of PNV diagnosis was significantly higher in UL-VWFM positive group than that in UL-VWFM negative group (*P* = 0.045). The rate of subretinal hemorrhage (*P* < 0.001) and PCV (*P* = 0.001) was also significantly higher in UL-VWFM positive group.

**Table 3:** The comparison of patients; UL-VWFM (+) and UL-VWFM (−).

	UL-VWFM (+) (n = 23)	UL-VWFM (−) (n = 74)	*P* value
Age, median (IQR)	77.0 (70.5–86.0)	77.0 (71.0–81.8)	0.44^†^
Sex (male), n (%)	20 (87)	50 (68)	0.11^‡^
BMI, median (IQR), kg/m^2^	20.2 (18.7–23.7)	22.2 (20.4–23.3)	0.32^†^
**Smoking status, n (%)**			
Never	6 (26)	39 (53)	–
Past or current	17 (74)	35 (47)	0.032^‡^
Current	6 (26)	22 (30)	0.80^‡^
**Blood type, n (%)**			
Type O	8 (35)	22 (30)	0.80^‡^
Type A	8 (35)	26 (35)	0.80^‡^
Type B	4 (17)	15 (20)	1^‡^
Type AB	3 (13)	11 (15)	1^‡^
PNV, n (%)	12 (52)	21 (28)	0.045^‡^
AMD, n (%)	11 (48)	53 (72)	–
Subretinal hemorrhage, n (%)	8 (35)	4 (5)	< 0.001^‡^
IRF, n (%)	4 (17)	12 (16)	1^‡^
SRF, n (%)	14 (61)	47 (64)	0.81^‡^
PED, n (%)	11 (48)	15 (20)	0.015^‡^
PCV, n (%)	17 (74)	25 (34)	0.001^‡^

*UL-VWFM* unusually large VWF multimer, *IQR* inter-quartile range, *PNV* pachychoroid neovasculopathy, *AMD* age related macular degeneration, *IRF* intra-retinal fluid, *SRF* subretinal fluid, *PED* pigment epithelium detachment, *PCV* polypoidal choroidal vasculopathy.

^†^Mann–Whitney U test.

^‡^ Fisher’s exact test.

**Figure2:**
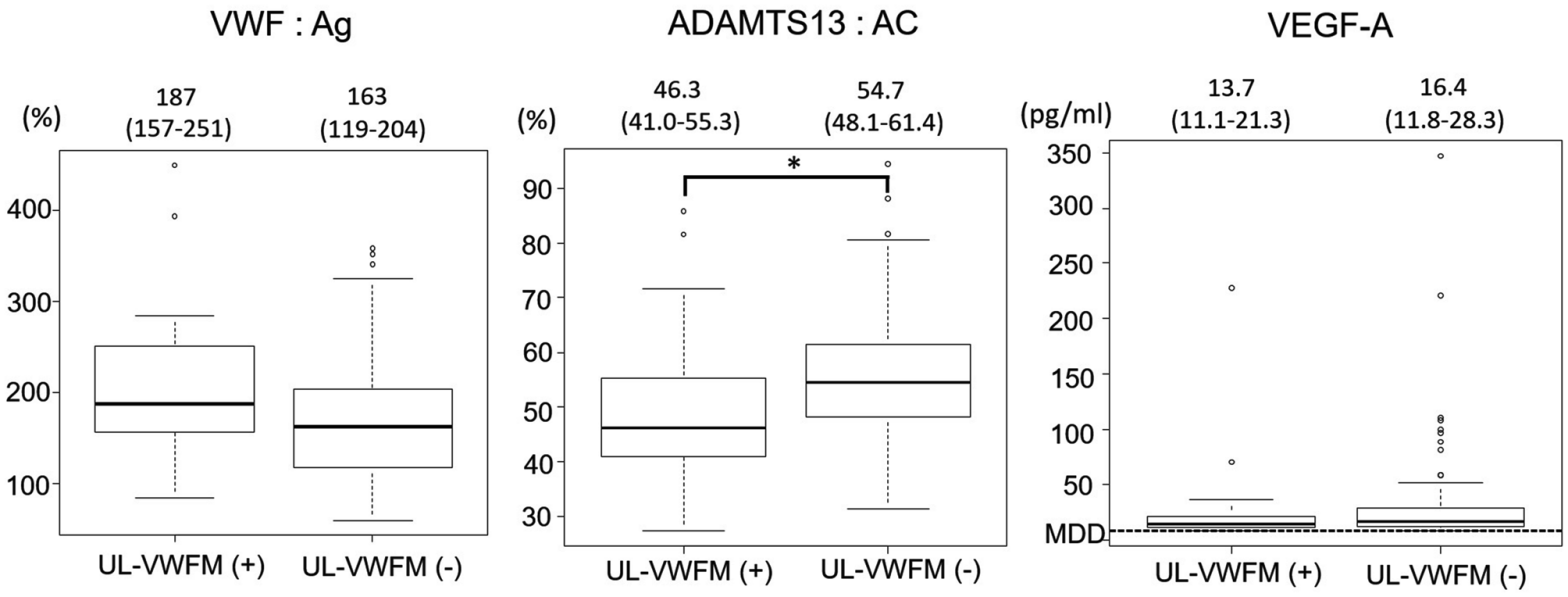
VWF: Ag, ADAMTS13:AC, and VEGF-A in UL-VWFM (+) and UL-VWFM (−). Each of the values in the graph is the median with the interquartile range. The ADAMTS13:AC was significantly lower in UL-VWFM (+) than in UL-VWFM (−) (*P < 0.05). VWF: Ag, plasma VWF: Antigen. ADAMTS13: AC, plasma ADAMTS13 activity. VEGF-A, vascular endothelial growth factor A.

ADAMTS13: AC was significantly lower in UL-VWFM positive group (*P* = 0.023). In contrast, there were no significant differences in VWF: Ag and VEGF-A between two groups (Fig. 2).

The distribution of gene polymorphisms in the *CFH* was showed in Tables 4 and 5. In the I62V, although there was no significant difference in the allele A frequency between PNV group and AMD group (OR 1.41; 95% CI 0.69–2.94, *P* = 0.44), the recessive homozygous (AA) was found only in PNV group. In the Y402H, the recessive homozygous (CC) did not exist in both groups and there was no significant difference in allele C frequency (OR 0.20; 95% CI 0.03–1.28, *P* = 0.17).

**Table 4:** The *CFH* I62V and Y402H gene polymorphisms in PNV and AMD.

	I62V			Y402H		
	AA	GA	GG	CC	TC	TT
PNV	4	7	22	0	1	32
AMD	0	22	42	0	9	55

**Table 5:** Odds ratio of each allele.

I62V	A	G	OR; A vs G (95% CI)	*P* value
PNV	15	51	1.41 (0.69–2.94)	0.44^†^
AMD​	22	106	–	

Y402H	C	T	OR; C vs T (95% CI)	*P* value
PNV	1	65	0.20 (0.03–1.28)	0.17^†^
AMD	9	119	–	

*CFH* complement factor H, *OR* odds ratio, *CI* confidence interval.

^†^Fisher’s exact test.

## Discussion

Recently, Hosoda et al. used machine learning to review Japanese patients with AMD and suggested that 46% of cases were reclassified as PNV^23^. However, the diagnosis criteria for PNV have not been clearly determined. Thus, we employed the definition criteria of previous reports^19,24^ and found a strong correlation between CCT and CVD. This suggests that the combination of both parameters in the diagnosis of PNV may lead to a more accurate assessment. In our study, 34% of cases were reclassified as PNV. This percentage was relatively lower than Hosoda’s report but still demonstrated that many cases previously diagnosed as AMD would be PNV by clinical characteristics.

Interestingly, our results showed the differences in clinical and biological characteristics between PNV and AMD. The rate of SRF was significantly higher in PNV group than in AMD group. This seems reasonable considering previous reports of PNV secondary to CSC^18,19^. The rate in the presence of UL-VWFMs was significantly higher in PNV group than in AMD group.

We also showed that subretinal hemorrhages were significantly higher in PNV group than in AMD group. Tagawa et al. reported that subretinal hemorrhages were found in 20% of patients with PNV^25^. Nagai et al. also revealed that the pachyvessels (CVD ≥ 180 μm) and pachychoroid (CCT ≥ 220 μm) are risk factors for macular exudative changes after the induction phase of anti-VEGF injections^24^. These findings suggested that the CNVs were more likely to proceed hemorrhage in eyes in PNV group than in AMD group. Our results also showed that subretinal hemorrhages and PCV were significantly higher in the UL-VWFM positive group. The increased incidence of other thrombotic disorders with bleeding in UL-VWFM-positive patients has been shown in previous studies^11,13,14^.

A recent study reported that the density of the choriocapillaris was decreased in the PNV, and the decreased density areas were corresponding to the presence of pachyvessels^26^. These results suggest that the pachyvessels may compress the choriocapillaris, then this leads to ischemia in the choroidal surface layer. Gelfand et al. have proposed a hemodynamic model of high shear stress acting on the choriocapillaris in eyes with AMD^27^. Matsumoto et al. reported the presence of anastomosis and formation of collateral blood vessels in the pachyvessels, suggesting a long-term blood stasis^28^. Thus, the vessels of the choriocapillaris in eyes with PNV would have especially higher shear stress than that in eyes with AMD.

It is known that the UL-VWFMs under low shear stress in large vessels take globular forms and are difficult to bind to platelets. However, in the micro vessels, UL-VWFMs are exposed to high shear stress and become extended forms that easily bind to platelets^11,29^. Normally, ADAMTS13 has an antithrombotic function by promptly cleaving UL-VWFMs which maintains the proper hemostasis. We found that ADAMTS13: AC was significantly lower in UL-VWFM positive group than that in UL-VWFM negative group. That means remained UL-VWFMs existed although ADAMTS13 was consumed. Notably, the results of recent studies indicated that CFH promotes the cleavage of UL-VWFMs^8–10^. That is, CFH specifically binds to the UL-VWFM A2 domain where located the cleavage site of ADAMTS13, then the UL-VWFM cleavage by ADAMTS13 is enhanced^8^. Together with these results, genetic alterations of *CFH* in PNV may lead to the persistence of UL-VWFMs and may induce platelet thrombosis.

Recently, Yamashiro et al. revealed that in *CFH* I62V, the G allele is a risk gene for developing AMD and a protective gene for developing PNV, while the A allele is a risk gene for developing PNV and a protective gene for developing AMD^3,20^. However, there was no significant difference in allele A frequency in *CFH* in our study, while the recessive homozygous (AA) was found only in PNV group. Because the number of patients was limited, the difference might not be significant. We also found that there is no significant difference between PNV and AMD in the Y402H.

The choriocapillaris in patients with PNV is supposed to be under high shear stress suppressed by the pachyvessels. This situation may further activate UL-VWFM leading to thrombus formation and the further deterioration of blood flow in the choriocapillaris. The deterioration of blood flow may promote CNV formation and make it more likely to bleeding, subretinal hemorrhage.

There are several limitations in this study. First, we used a relatively small sample size for the comparisons. Second, the long-term clinical course is still unknown because we evaluated only at the first examination. Further long-term follow-up is desired to determine the clinical differences of the two groups.

## Conclusions

The residual UL-VWFMs in the plasma may result in the formation of platelet thrombosis and hemorrhages in the choriocapillaris of PNV. Our findings suggest biological differences between PNV and AMD.

## Methods

This was a cross-sectional study of 97 treatment naïve patients who had been diagnosed with exudative AMD based on the definition of the Japanese AMD Study Group^30^. In more detail, exudative AMD was diagnosed in patients who were over 50 years old with at least one following abnormalities (CNV, serous pigment epithelium detachment (PED), subretinal hemorrhage, and fibrous scars) within an area of 6000 μm in diameter centered at the fovea by using fundus photography, optical coherence tomography (OCT), fluorescein angiography (FA), and indocyanine green angiography (ICGA). Severe myopia (over − 6.0 diopter), uveitis, trauma and other degenerative diseases were excluded. All participants were patients of the Nara Medical University Hospital, Kashihara City, Nara Prefecture, Japan from 2014 to 2019. This study protocol was approved by the Institutional Review Board of the Nara Medical University and a signed informed consent was obtained from all the participants before the examinations. This study was performed in accordance with the Declaration of Helsinki. We confirmed that all methods were performed in accordance with the relevant guidelines and regulations.

All patients underwent conventional ophthalmological examinations including slit-lamp examinations, fundus examinations, OCT (Spectralis, Heidelberg Engineering, Dossenheim, Germany), fundus photography, FA, and ICGA at the initial examination. All of the abnormalities, such as subretinal hemorrhages, intra- or subretinal fluid, PED, and polypoidal lesion were detected at that examination. The fundus photographs, OCT and FA images were used to determine the presence of subretinal hemorrhages by two retinal specialists. We used enhanced-depth imaging (EDI) OCT to increase the visibility of choroidal structures. Central choroidal thickness (CCT) and choroidal vessel diameter (CVD) were determined by EDI-OCT images. Type 1 CNV was defined as early leakage in FA and highly reflective lesions beneath the RPE by EDI-OCT. After re-examination of all of the images, the patients were diagnosed with PNV or AMD.

**Figure 3:**
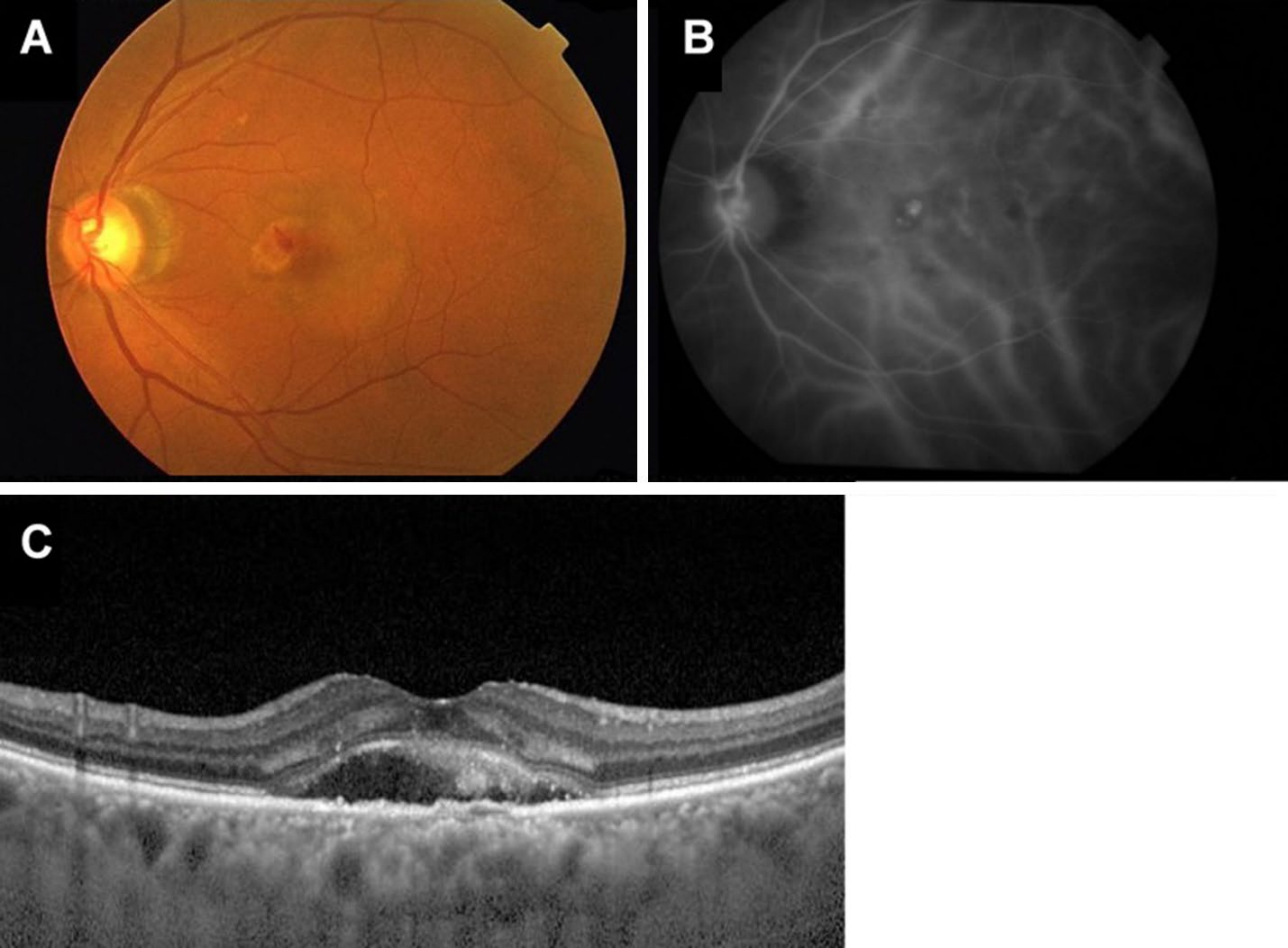
Fundus photograph of 76-year-old man with pachychoroid neovasuculopathy (PNV). (**A**) Drusen are not present in the fundus photograph. (**B**) Indocyanine green angiography (ICGA) showing increased choroidal vascular permeability. The dilated blood vessels pass through the areas close to the macula. (**C**) The central choroidal thickness (CCT) is 335 μm and the choroidal vessel diameter (CVD) is 201 μm. Choroidal vessels in Haller's layer are dilated.

According to the recent reports on PNV analysis^3,19,23–25,31^, we diagnosed a patient as PNV when all of the following criteria were satisfied: Type1 CNV (including PCV), CVD ≥ 180 μm, CCT ≥ 220 μm, absence of drusen in the fundus photographs, and increased choroidal vascular permeability in the late phase of the ICGA images (Fig. 3). PCV was characterized by polypoidal lesions in IGCA. CVD was defined as the vertical diameter of the largest vessel in the Haller's layer. CVD and CCT were measured independently by two different researchers using a scale bar contained within the OCT system^24^. The judgments were made independently by two retinal specialists, and in cases when the judgments were different, the final decision was made by a macular disease specialist (N.O.).

**Figure 4:**
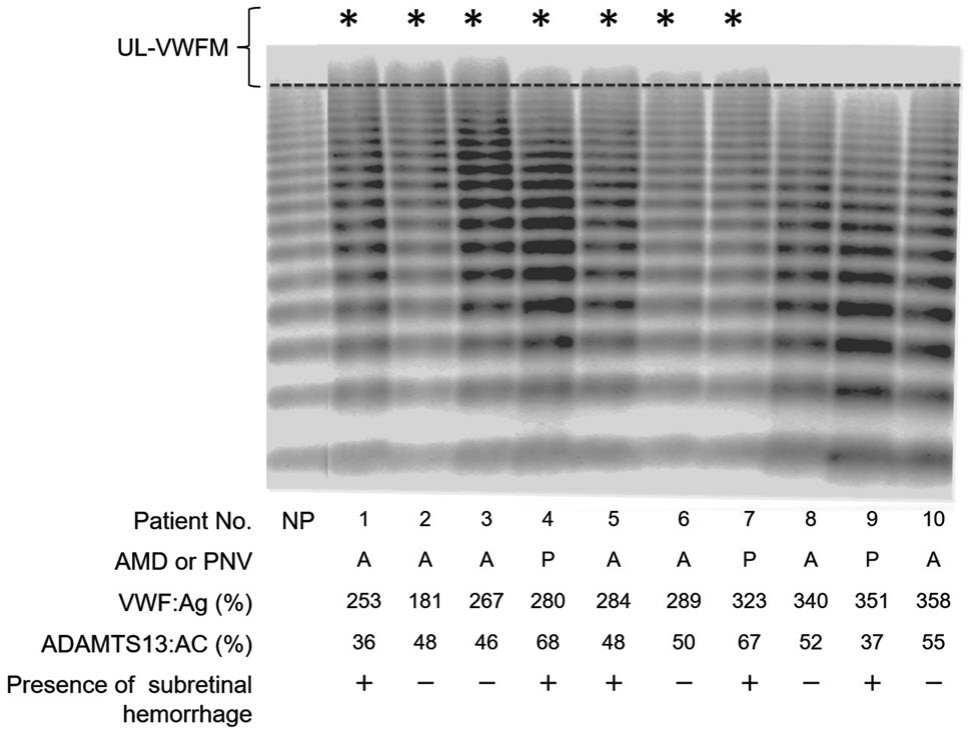
von Willebrand factor (VWF) multimer analysis in representative cases. VWF multimer of normal pool plasma from 40 healthy subjects (NP) is shown in the far left column, and the plasma of representative 10 cases are the next 10 columns. The VWF multimers above the dotted line are defined as unusually large VWF multimers (UL-VWFMs). AMD, age-related macular degeneration; PNV, pachychoroid neovasculopathy. VWF: Ag, plasma VWF antigen. ADAMTS13: AC, plasma ADAMTS13 activity.

We collected blood samples by venipuncture at the initial examination. The whole blood samples were stored in tubes containing a 1:9 volume of 3.8% trisodium citrate. The plasma was separated and saved at – 80 °C. For the measurements, the plasma was thawed and maintained at 37 °C before the measurements. The level of plasma VWF Antigen (VWF: Ag) was measured by sandwich enzyme-linked immunosorbent assay (ELISA) using a rabbit anti-human VWF polyclonal antiserum (DAKO, Glostrup, Denmark)^14^. We determined the activity of ADAMTS13 (ADAMTS13: AC) by a chromogenic ADAMTS13 activity ELISA kit (Kainos, Tokyo, Japan)^32^. The 100% reference value was defined as the amount of VWF: Ag and ADAMTS13: AC in the plasma of 20 normal volunteers (10 men and 10 women ages 20–40 years). We also measured the plasma vascular endothelial growth factor A (VEGF-A) by ELISA (Quantikine VEGF ELISA kit; R&D Systems, Minneapolis, Minnesota, USA). The VWF multimers were analyzed by the method of Ruggeri and Zimmerman^33^, modified by Warren et al.^34^. The measurements were made under the conditions described by Budde et al.^35^ and we defined the high molecular weight bands that were not detected in normal plasma as UL-VWFMs. Then, we performed densitometric analysis using ImageJ (National Institute of Health, Bethesda, Maryland, USA). UL-VWFMs were defined as being positive when the ratio of UL-VWFMs to total VWF was > 1% (Fig. 4)^36^.

We extracted genomic DNA from the leukocytes by a genomic DNA kit (QIAmpDNA; Qiagen, Valencia, California, USA) and genotyped the SNPs of p.I62V (rs800292) and p.Y402H (rs1061170) in *CFH*. Polymerase chain reaction (PCR) with specific primers was used to amplify the polymorphic sites^37^. The PCR products was used as the templates for direct DNA sequencing (Applied Biosystems, Foster City, California, USA) on an automated sequencer (3730xl DNA analyzer; Applied Biosystems). The genotypes in the *CFH* were classified based on the previous studies^38,39^.

### Statistical analyses

Two-tailed Mann–Whitney U tests were used to compare the averages of continuous variables (such as age) and Fisher’s exact tests to compare the proportions of categorical variables (such as sex) between groups. Odds ratios and 95% confidence intervals were calculated by using Fisher’s exact tests. All statistical analyses were performed with EZR (Saitama Medical Center, Jichi Medical University, Saitama, Japan), which is a graphical user interface for R (The R Foundation for Statistical Computing, Vienna, Austria)^40^. Since both CCT and CVD followed a normal distribution, the correlation was evaluated at Pearson's correlation coefficient. On the other hand, the measured values of VEGF-A were not normally distributed, and the exact values were not partially shown because the values were below the minimum detectable dose of 9 pg/ml. Therefore, a non-parametric test was applied, and a conservative value of 8 pg/ml was input for each value below the MDD when performing the calculations. The threshold for statistically significance was *P* < 0.05.

## Data Availability

The datasets generated or analyzed during the current study are available from the corresponding author on reasonable request.
